# Glycomic Insights in Gynecological Disease: From Molecular Mechanisms to Precision Diagnostics and Therapeutics

**DOI:** 10.3390/ijms27031490

**Published:** 2026-02-03

**Authors:** Róbert Pásztor, Csaba Váradi

**Affiliations:** 1 Institute of Chemistry, Faculty of Materials Science and Chemical Engineering, University of Miskolc, 3515 Miskolc, Hungary; csuko67@gmail.com; 2 Borsod-Abaúj-Zemplén County Center Hospital and University Teaching Hospital, 3526 Miskolc, Hungary

**Keywords:** glycomics, gynecological cancer, biomarkers, molecular diagnostics, post-translational modifications, precision medicine, women’s health

## Abstract

Gynecological diseases—encompassing polycystic ovary syndrome, endometriosis, infertility, and malignancies—represent a significant global health burden affecting women’s quality of life, reproductive capacity, and long-term health outcomes. While traditional diagnostics rely on protein-based biomarkers, clinical phenotyping, and imaging, these approaches often lack the sensitivity and specificity required for early detection and personalized intervention. Glycomics, the comprehensive study of carbohydrate structures on proteins and lipids, represents an emerging molecular frontier in gynecological disease characterization and therapeutics. This review synthesizes current knowledge regarding glycomic dysregulation across gynecological conditions, elucidates how aberrant glycosylation patterns serve as disease-specific biomarkers, and demonstrates key translational applications, such as glycoform-specific CA-125. By integrating glycomics with complementary omics technologies and artificial intelligence-driven analysis, a transformative diagnostic paradigm is emerging that promises earlier detection, improved risk stratification, and precision-guided therapeutics for women with gynecological disorders.

## 1. Introduction

### 1.1. The Clinical Challenge in Gynecological Disease Management

Women’s gynecological health encompasses a complex spectrum of conditions ranging from metabolic and endocrine disorders to inflammatory diseases and malignancies [1]. Polycystic ovary syndrome (PCOS) affects 8–20% of reproductive-age women, disrupting metabolic homeostasis and reproductive function through dysregulated insulin signaling and hyperandrogenism [2]. Endometriosis, characterized by ectopic endometrial growth, affects 10–15% of reproductive-age women and is frequently misdiagnosed, with diagnostic delays averaging 7–10 years [3]. Gynecologic malignancies—ovarian, endometrial, and cervical cancers—collectively account for approximately 16.1% of new cancer diagnoses in women globally, with over 680,000 deaths annually, largely attributable to late-stage detection [4]. The fundamental challenge traversing all of these conditions is the inadequacy of current diagnostic biomarkers. For gynecologic malignancies, standard markers such as CA-125 (MUC16) and HE4 demonstrate sensitivity for early-stage disease of only 50–76% while generating substantial false positives in benign conditions [5]. For PCOS and endometriosis, diagnosis remains primarily clinical and imaging-based, lacking robust molecular biomarkers that could facilitate earlier intervention and personalized treatment selection [6]. These diagnostic limitations are compounded by the increasing recognition that traditional single-protein biomarkers capture insufficient biological information to distinguish disease states from healthy conditions or to predict individual therapeutic responsiveness.

### 1.2. The Glycomic Revolution: From Biochemistry to Clinical Translation

Glycosylation—the enzymatic attachment of carbohydrate chains (glycans) to proteins and lipids—represents the most abundant and chemically diverse post-translational modification in human cells, yet remains underutilized in clinical diagnostics [7]. More than 50% of human proteins undergo glycosylation, and remarkably, nearly all FDA-approved cancer biomarkers are glycoproteins [8]. The critical insight driving the glycomic revolution is that cancer cells and cells in pathological states exhibit systematic dysregulation of glycosylation enzymes (glycosyltransferases and glycosidases), resulting in characteristic carbohydrate modifications absent from healthy tissue [9]. These glycomic “signatures” represent a previously unexploited reservoir of diagnostic and therapeutic information that could transform precision medicine approaches across gynecological diseases [10].

Recent technological advances in mass spectrometry, artificial intelligence, and multi-omics integration have made glycomic analysis increasingly feasible for clinical implementation [11]. Techniques such as HILIC-UPLC-MS/MS, combined with machine learning algorithms and integrated with genomic, transcriptomic, and proteomic data, now enable the identification of glycomic signatures with unprecedented sensitivity and specificity [12]. This review synthesizes emerging evidence regarding glycomic dysregulation across gynecological diseases and demonstrates how glycomic biomarkers and glycan-targeting therapeutics represent a transformative approach to women’s health. Therefore, biomarkers and glycan-targeting therapeutics represent a transformative approach to women’s health. This review also identifies a distinct “metabolic–glycomic axis” that differentiates non-malignant conditions from cancers. While malignant glycomes are typically reshaped by epigenetic silencing of chaperones (e.g., *COSMC*) or enzymatic reprogramming, the glycomic defects in PCOS and metabolic syndrome appear to be driven by substrate availability. The shift toward enhanced glycolysis in these patients alters the pool of nucleotide sugar donors, which systematically reshapes the serum glycome. This mechanistic distinction is critical for future research as it suggests that therapeutic interventions in metabolic gynecological disorders might focus on metabolic normalization to correct the glycome, whereas malignancies require the direct targeting of aberrant glycans or the immune checkpoints they engage. To facilitate a comprehensive understanding of this complex molecular landscape, Table 1 synthesizes the primary glycomic alterations observed across gynecological pathologies, distinguishing between the mechanisms driving malignancies and those underlying metabolic or inflammatory conditions.

## 2. Molecular Mechanisms: Glycosylation Dysregulation in Gynecological Diseases

### 2.1. Biochemical Foundations of N-Linked and O-Linked Glycosylation

Glycosylation in eukaryotic cells proceeds through two primary pathways that differ in initiation site and subcellular localization [13]. N-linked glycosylation initiates in the endoplasmic reticulum with transfer of a preformed high-mannose glycan to asparagine residues within consensus sequences (Asn-X-Ser/Thr), followed by sequential processing in the Golgi apparatus to generate complex-type N-glycans with diverse branching architectures [14]. The biosynthetic precision of N-glycosylation is governed by expression levels and catalytic activities of approximately 100 distinct N-glycan processing glycosyltransferases and glycosidases, creating a delicate equilibrium that distinguishes healthy from pathological states [15].

O-linked glycosylation, encompassing mucin-type modifications where glycans attach to serine and threonine residues, begins with transfer of N-acetylgalactosamine (GalNAc) by peptidyl-N-acetylgalactosaminyltransferases (GALNTs) in the Golgi apparatus, followed by optional extension through additional enzymatic steps [16]. In healthy cells, O-glycans undergo orderly maturation through coordinated enzymatic action; in pathological states, this process is characteristically interrupted at early steps, producing truncated structures (Tn antigens and sialyl-Tn) that serve as disease biomarkers [17].

### 2.2. Glycomic Dysregulation in Gynecologic Malignancies

In ovarian, endometrial, and cervical cancers, aberrant glycosylation manifests through multiple converging mechanisms [10]. Enhanced branching of N-glycans through upregulation of GnT-V (encoded by MGAT5) creates structures that bind galectin-3, forming surface lattices that sequester growth factor receptors and drive constitutive proliferative signaling [18]. Hypersialylation—excessive terminal sialic acid residues—creates a “don’t eat me” glycan shield that engages Siglec receptors on immune cells, delivering inhibitory signals that suppress anti-tumor immunity [19]. Truncation of O-glycans, driven by epigenetic silencing of COSMC (a critical chaperone for O-glycan maturation), exposes tumor-associated carbohydrate antigens (TACAs) including sialyl-Tn (sTn), which are absent from healthy tissues but prevalent in gynecologic malignancies [20].

### 2.3. Glycosylation Dysregulation in PCOS and Metabolic Dysfunction

Polycystic ovary syndrome is characterized by insulin resistance, hyperandrogenism, and systemic inflammation, all of which are intimately linked to metabolic dysregulation and altered protein post-translational modifications [21]. The dysregulated glucose and lipid metabolism characteristic of PCOS results in altered availability of nucleotide sugar substrates (UDP-glucose, UDP-GlcNAc, CMP-sialic acid) used for glycosylation biosynthesis, thereby systematically reshaping the glycomic landscape of serum glycoproteins and reproductive tract tissues [22]. Specifically, enhanced glycolysis in PCOS shifts biosynthetic precursor pools toward glycolytic intermediates and away from oxidative pathways, creating a metabolic environment that favors particular glycan structures over others. Inflammatory cytokines elevated in PCOS (TNF-α, IL-6) activate inflammatory transcription factors that upregulate sialyltransferases and other glycan-modifying enzymes, resulting in elevated sialylation of circulating proteins [23]. These PCOS-associated glycomic signatures could serve as biomarkers for disease identification and stratification of disease severity, enabling earlier intervention and more precise therapeutic targeting [24].

### 2.4. Glycosylation Alterations in Endometriosis and Endometrial Pathology

Endometriosis is characterized by dysregulated estrogen signaling, elevated local inflammation, and altered endometrial receptivity, all processes intimately linked to protein modifications including glycosylation [25]. Estrogen receptor alpha (ESR1) and its coregulators, including ZMIZ1, control the expression of genes encoding glycosylation enzymes through transcriptional regulatory mechanisms [26]. In endometriosis, dysregulated estrogen signaling and reduced ZMIZ1 expression result in altered glycosylation patterns on proteins critical for cell-cell adhesion, immune cell recognition, and endometrial receptivity [27]. The identification of glycomic signatures specific to eutopic endometrium from women with endometriosis—distinguishing it from healthy controls—could facilitate non-invasive diagnosis and enable earlier intervention before ectopic lesion establishment [28].

## 3. Glycomic Biomarkers: Diagnostic Applications and Clinical Translation

### 3.1. Glycoform-Specific Biomarkers for Cancer Early Detection

The most clinically advanced glycomic biomarker application involves measuring glycan modifications on established cancer-associated proteins rather than developing entirely novel markers [29]. Recent 2024–2025 studies demonstrate that specific fucosylated and sialylated glycoforms of CA-125 achieve diagnostic accuracy for ovarian cancer exceeding 95% specificity, substantially surpassing the ~70% specificity of total CA-125 measurement [30]. This glycoform-specific approach represents a paradigm shift: rather than measuring crude protein abundance, clinicians assess the carbohydrate “language” encoded on these proteins, which differs characteristically between cancer and benign sources [31]. Cancer-derived CA-125 carries distinctive patterns of sialylation, fucosylation, and N-glycan branching that reflect the dysregulation of glycosyltransferases in tumor cells, creating recognizable molecular signatures absent from CA-125 produced by healthy endometrial or epithelial cells [32]. Traditional protein-only biomarkers, such as CA-125, often fail to distinguish between malignancy and benign inflammation, leading to high false-positive rates and unnecessary invasive procedures. The data presented here suggests that the solution lies in qualitative rather than quantitative analysis. By measuring the “carbohydrate language” of these proteins—specifically the patterns of hypersialylation and fucosylation—clinicians can achieve a level of specificity previously unattainable, distinguishing cancer-derived glycoproteins from their benign counterparts with high precision.

Clinically, implementation of glycoform-specific biomarkers could function through reflex testing: when CA-125 is elevated using current thresholds, the sample is automatically analyzed for cancer-specific glycoforms [33]. If present, the probability of malignancy is high, warranting imaging and possible surgical intervention. If absent, the elevation likely reflects benign disease, avoiding unnecessary procedures. This simple addition to existing workflow infrastructure could dramatically reduce diagnostic uncertainty, unnecessary procedures, and patient anxiety while simultaneously improving cancer detection rates in early, more treatable stages [30]. Beyond free circulating glycoproteins, recent attention has shifted toward the glycomic profiling of extracellular vesicles (EVs), particularly exosomes. These membrane-bound nanovesicles secreted by tumor cells carry a unique molecular cargo that mirrors the parental cell’s status, including surface proteins with distinct tumor-associated glycosylation patterns [34]. In gynecological malignancies, specific alterations in N- and O-glycosylation on EV surfaces have emerged as critical diagnostic targets. For instance, ovarian cancer-derived exosomes have been shown to be enriched with specific sialoglycoproteins, such as Galectin-3-binding protein (LGALS3BP), and exhibit unique N-glycan signatures characterized by increased bisecting GlcNAc and α2,3-sialylation compared to benign vesicles [35]. These vesicular glyco-profiles offer a ‘concentrated’ source of tumor-specific markers, potentially overcoming the dilution and specificity issues inherent in analyzing whole serum [36].

### 3.2. Integrated Multi-Omics Biomarker Panels

The future of gynecological disease diagnostics lies in integrated panels combining glycomic, genomic, epigenomic, and metabolomic data within machine learning frameworks [37]. For gynecologic malignancies specifically, integrated biomarker panels might combine: (1) glycoform-specific CA-125 and HE4 measurements; (2) circulating tumor DNA (ctDNA) analysis revealing tumor-specific mutations and copy number alterations; (3) circulating tumor cell (CTC) enumeration and characterization; (4) extracellular vesicle-derived glycosylated exosomal microRNAs; and (5) circulating free DNA methylation patterns reflecting epigenetic changes [38,39]. Machine learning algorithms trained on prospective cohorts can integrate these multi-dimensional data to generate predictive models with a sensitivity and specificity far exceeding any single marker, achieving the diagnostic precision necessary for population-based screening or high-risk population surveillance.

For PCOS diagnosis, integrated panels could combine classical biochemical parameters (testosterone, LH/FSH ratios) with genomic variants identified through genome-wide association studies (such as WNT4, ESR1, FSHB polymorphisms), with epigenetic biomarkers (DNA methylation patterns, miRNA expression profiles), and with novel glycomic signatures reflecting the metabolic and inflammatory state [40]. The OvAge algorithm, which integrates clinical, hormonal, and ultrasound parameters into a single output, exemplifies the successful integration of multiple data types to improve diagnostic accuracy and could be expanded to incorporate glycomic and other molecular data [41].

### 3.3. Glycomic Signatures in Endometrial Receptivity and Implantation Competence

Uterine receptivity—the capacity of the endometrium to accept embryo implantation—is a precisely regulated physiological state controlled by estrogen and progesterone signaling through coordinated transcriptional and post-translational modifications of endometrial proteins [42]. Emerging evidence suggests that glycosylation of adhesion molecules, growth factors, and cytokines plays critical roles in regulating embryo–endometrial interactions essential for successful implantation [43]. Dysregulation of glycosylation patterns in the endometrium of women with recurrent implantation failure or recurrent pregnancy loss could serve as diagnostic biomarkers enabling the identification of women who would benefit from specific interventions to correct endometrial glycosylation defects [44].

The glycosylation of follistatin, a critical regulator of uterine receptivity through modulation of TGF-β family signaling, represents one example of glycomic involvement in reproductive competence [45]. Glycoform-specific quantification of follistatin and related molecules in serum, endometrial tissue, or follicular fluid could identify women at risk for implantation failure and could potentially guide targeted interventions to normalize glycosylation patterns [46]. Non-invasive diagnostic tests measuring endometrial glycomic signatures through liquid biopsy (circulating endometrial cells, extracellular vesicles, cfDNA methylation) could enable the preimplantation assessment of receptivity without invasive endometrial biopsy, transforming assisted reproductive technology and fertility preservation strategies [47].

## 4. Technological Integration: Glycomics Within the Multi-Omics Landscape

### 4.1. Mass Spectrometry-Based Glycomic Profiling and Analytical Validation

Modern glycomic analysis relies upon high-resolution mass spectrometry techniques that enable the identification and quantification of individual glycan structures with extraordinary precision. HILIC-UPLC-MS/MS achieves the separation of glycan isomers—molecules with identical chemical formulas but different three-dimensional structures—a distinction critical since isomers frequently possess different biological functions and different associations with disease [48]. Matrix-assisted laser desorption/ionization mass spectrometry imaging (MALDI-MSI) enables the spatial mapping of glycan structures within tissue sections, revealing that specific glycan classes accumulate in particular tumor microenvironmental compartments such as cancer-associated fibroblasts, immune cell infiltrates, or hypoxic regions [49]. These spatial glycomic profiles provide insights into the glycomic landscape of the tumor microenvironment that could inform therapeutic targeting and predict immunotherapy responsiveness provide insights into the glycomic landscape of the tumor microenvironment that could inform therapeutic targeting and predict immunotherapy responsiveness [50].

The analytical standardization of glycomic assays remains an ongoing challenge requiring the development of standardized protocols, reference materials, proficiency testing programs, and harmonized data interpretation criteria [51]. Professional societies and consortia are establishing these standards, facilitating the translation of research-stage glycomic methods into clinically validated diagnostic tests. Quality control measures including instrument standardization, reagent lot standardization, and operator training will be essential for ensuring reproducibility across clinical laboratories [52].

### 4.2. Artificial Intelligence-Driven Glycomic Data Interpretation

The complexity of glycomic datasets—with hundreds to thousands of distinct glycan structures potentially present in a single sample—necessitates sophisticated computational approaches for pattern recognition and biological interpretation [53]. Machine learning algorithms including deep neural networks, random forests, support vector machines, and ensemble methods can identify multidimensional glycomic signatures predictive of disease state, disease severity, treatment response, or clinical outcomes [54]. Recent applications of convolutional neural networks (CNNs), particularly for imaging-based biomarker analysis using Raman spectroscopy combined with AI, have achieved diagnostic accuracies exceeding 95% for various cancer types, with similar applications emerging for gynecologic malignancies [55].

Critical to clinical adoption is the development of explainable AI approaches wherein the specific glycan features driving predictions are transparent to clinicians [56]. Black-box algorithms that achieve high accuracy but cannot be interpreted by clinicians will face substantial barriers to clinical implementation [57]. Rather, interpretable machine learning models that identify biologically meaningful glycomic patterns—for instance, elevated fucosylation specifically at core N-glycan positions combined with reduced sialylation on O-glycans—will enable clinical understanding and support integration into clinical decision-making workflows [58].

### 4.3. Integration with Genomic and Epigenomic Data

The recognition that cancer and gynecological diseases result from convergent dysregulation of multiple biological layers—genomic, epigenomic, transcriptomic, proteomic, and glycomic—has catalyzed the development of integrated multi-omics approaches that simultaneously measure mutations, epigenetic modifications, RNA expression, protein levels, and glycosylation patterns [59]. For instance, specific genomic mutations in gynecologic cancers (such as BRCA1/2 mutations, PTEN loss, or TP53 mutations) are associated with characteristic glycomic signatures reflecting altered metabolic state and signaling pathway activation downstream of these mutations [60]. Epigenetic modifications including DNA methylation patterns and histone post-translational modifications regulate the expression of glycosyltransferases and glycosidases, thereby controlling glycomic output [61]. Integration of these multi-layer datasets within machine learning frameworks enables the identification of convergent molecular signatures with substantially greater predictive power than any single data layer alone [62].

## 5. Therapeutic Applications: Targeting Glycomic Dysregulation

The convergence of diagnostic targets and therapeutic opportunities creates a unique “theranostic” landscape in women’s health. The same Sialyl-Tn (sTn) and Tn antigens that serve as highly specific biomarkers for ovarian and endometrial cancers are now being leveraged as targets for next-generation antibody–drug conjugates (ADCs) and CAR-NK therapies. Because these truncated O-glycans are essentially absent from healthy tissues, they offer a “narrow-spectrum” therapeutic window that could significantly reduce the off-target toxicities associated with conventional chemotherapy. However, despite this promise, significant hurdles remain. The complexity of the glycome requires a multidisciplinary approach, combining high-resolution analytical chemistry with clinical oncology to ensure that these glycan-targeted strategies can be standardized for routine clinical use.

### 5.1. Antibody–Drug Conjugates and CAR-T Cell Therapy Targeting Glycan Epitopes

The same glycan structures that define disease through diagnostic biomarkers can be therapeutically targeted through immunotherapy approaches [63]. Antibody–drug conjugates (ADCs) directed against tumor-associated carbohydrate antigens (TACAs) such as sialyl-Tn (sTn) combine antibody-mediated specificity for tumor-restricted glycan epitopes with cytotoxic payloads, enabling the selective elimination of cancer cells while sparing healthy tissue. sTn-targeting ADCs are currently entering clinical trials for ovarian, endometrial, and cervical cancers, with preliminary data suggesting activity in disease resistant to conventional chemotherapy [64]. The theoretical advantage of sTn-targeting ADCs lies in the exquisite tumor selectivity: sTn is essentially absent on normal tissues yet abundant on gynecologic malignancies, potentially enabling therapeutic efficacy with substantially reduced off-target toxicity compared to conventional cytotoxic chemotherapy [65].

Chimeric antigen receptor (CAR)-engineered T cells and natural killer (NK) cells targeted against glycopeptide epitopes represent another rapidly advancing therapeutic platform [66]. CAR-NK cells targeting Tn-MUC1 (mucin-1 proteins carrying incomplete O-glycosylation with exposed Tn antigens) have demonstrated preliminary efficacy in early trials, with the advantage that engineered NK cells can be derived from allogeneic donors, potentially enabling “off-the-shelf” therapeutic products without requiring individualized manufacturing [67]. CAR-T cells and CAR-NK cells targeting glycopeptides combine the adoptive cellular immunity advantages of these platforms with the tumor selectivity conferred by glycan-specific targeting, offering potential for durable anti-tumor responses and possible long-term immune memory (Table 2) [68].

### 5.2. Glycan-Targeting Immunotherapy and Sugar-Stripping Enzymes

Hypersialylation of tumor-derived proteins creates a “don’t eat me” glycan shield through engagement of Siglec receptors on immune cells, suppressing anti-tumor immunity through mechanisms functionally equivalent to protein-based checkpoint inhibition. Sugar-stripping enzymes, particularly sialidase fusion proteins that enzymatically remove terminal sialic acid residues, are currently entering clinical trials with the objective of unmasking hidden tumor antigens and eliminating glycan-mediated immune suppression [69]. The combination of sialidase-mediated glycan removal with conventional checkpoint inhibitors targeting protein-based checkpoints (such as anti-PD-1 antibodies) represents a rational therapeutic strategy targeting immune suppression through two complementary mechanisms [70]. Preclinical data support this concept, demonstrating synergistic anti-tumor activity when sialidase is combined with checkpoint inhibitors in multiple cancer models including gynecologic malignancies [71].

### 5.3. Glycosyltransferase and Glycosidase Inhibition

Direct inhibition of glycosyltransferases or glycosidases represents another therapeutic avenue for targeting glycomic dysregulation [72]. For instance, selective inhibition of sialyltransferases such as ST6GALNAC4, which is upregulated in many cancers through MYC-driven transcriptional activation, could reduce the hypersialylation that drives immune evasion [73]. Similarly, inhibitors of fucosyltransferases could reduce fucosylation-driven epithelial-to-mesenchymal transition and metastatic dissemination in cervical and other adenocarcinomas [74]. The therapeutic challenge lies in achieving sufficient selectivity to avoid disrupting the glycosylation of normal proteins in healthy tissues, potentially limiting clinical applicability [75]. However, the discovery that specific glycosyltransferases are preferentially upregulated in particular tumor types or are driven by specific oncogenic pathways (such as MYC-driven ST6GALNAC4 upregulation) offers opportunities for relative selectivity. Combination strategies pairing glycosyltransferase inhibition with conventional chemotherapy or immunotherapy could potentially improve outcomes by reducing glycan-mediated immune suppression while simultaneously treating the primary tumor [76].

## 6. Conclusions and Future Perspectives

The evidence synthesized in this review highlights a fundamental paradigm shift: glycosylation is not merely a secondary post-translational modification but a dynamic, functional driver of gynecological disease. By examining the spectrum from metabolic disorders like PCOS to lethal malignancies, it becomes clear that glycomic alterations are “functionally consequential”. Rather than serving as passive bystanders, glycans actively facilitate disease progression by orchestrating immune evasion and hyper-activating growth signaling.

### 6.1. Regulatory Pathways and Clinical Validation Requirements

Translation of glycomic discoveries into clinical diagnostics and therapeutics faces several regulatory and validation hurdles [77]. Novel diagnostic tests require the demonstration of analytical validity (accurate and reproducible measurement of glycan structures), clinical validity (glycan biomarkers correctly identify disease), and clinical utility (use of glycan biomarkers improves patient outcomes) [78]. Large prospective clinical studies enrolling diverse populations—healthy controls, patients with benign gynecologic conditions, and patients with various gynecologic diseases at different stages—are essential for rigorous validation [79]. Independent validation cohorts must demonstrate that glycomic signatures identified in training cohorts generalize to independent populations, a critical requirement that current preliminary studies have not yet rigorously addressed [80].

Regulatory approval pathways differ by jurisdiction but generally involve either the development of laboratory-developed tests (LDTs) under Clinical Laboratory Improvement Amendments (CLIA) certification in the United States or approval through the FDA as in vitro diagnostic devices [81]. The glycomic field currently lacks consensus on which regulatory pathway is most appropriate, with ongoing discussions among diagnostic companies, academic laboratories, and regulatory agencies regarding optimal approaches to enable timely clinical translation while ensuring analytical rigor and patient safety [82].

### 6.2. Health Equity and Access Considerations

Current glycomic analysis using high-resolution mass spectrometry requires sophisticated instrumentation and specialized expertise available only in advanced academic centers and diagnostic companies, raising substantial concerns regarding health equity and global access [83]. Development of simplified, point-of-care glycomic tests—potentially using novel biosensor technologies, simplified immunoassay approaches, or microfluidic platforms—could democratize access to these diagnostics and enable their implementation in resource-limited healthcare settings. International collaboration and technology transfer initiatives will be essential for ensuring that glycomic advances benefit women globally rather than primarily benefiting populations with access to advanced diagnostic centers [84].

### 6.3. Prospective Clinical Studies and Research Initiatives

Several large prospective clinical research initiatives are currently underway or planned to comprehensively evaluate glycomic biomarkers in gynecologic diseases. The National Centers for Translational Research in Reproduction and Infertility (NCTRI) represent one such initiative, bringing together multidisciplinary teams of researchers focused on reproductive medicine, including the investigation of molecular mechanisms and biomarkers in endometriosis, infertility, and related conditions. Academic cancer centers are establishing prospective biomarker trials collecting blood and tissue samples with comprehensive glycomic profiling combined with detailed clinical follow-up, enabling the correlation of glycomic signatures with treatment response, survival outcomes, and disease progression patterns. These initiatives recognize that rigorous validation in prospective cohorts is a prerequisite for clinical translation and that multidisciplinary collaboration across oncology, reproductive medicine, bioinformatics, and molecular biology is essential for success translation and that multidisciplinary collaboration across oncology, reproductive medicine, bioinformatics, and molecular biology is essential for success.

## 7. Conclusions

Glycomics represents an emerging frontier in molecular diagnostics and therapeutics for gynecological diseases, offering unprecedented opportunities to improve women’s health through earlier detection, enhanced risk stratification, and personalized therapeutic targeting. The recognition that cancer cells and cells in pathological gynecologic states exhibit systematic dysregulation of glycosylation machinery, resulting in characteristic carbohydrate modifications absent from healthy tissue, has opened entirely new diagnostic and therapeutic avenues. Glycoform-specific biomarkers for established proteins achieve diagnostic specificity and sensitivity substantially exceeding protein concentration-based testing, promising to transform early cancer detection and reduce unnecessary procedures in women with benign disease. Integration of glycomic data with complementary omics technologies—genomic, epigenomic, transcriptomic, proteomic—and artificial intelligence-driven analysis creates a multi-dimensional molecular characterization of disease, enabling precision medicine approaches tailored to individual biology.

Therapeutically, glycan-targeting strategies including antibody–drug conjugates, CAR-T cell therapy, and sugar-stripping enzymes represent genuinely novel mechanisms with activity in treatment-resistant disease. These approaches complement conventional therapies by targeting distinctive features of cancer and diseased cells—their aberrant glycosylation—offering potential for improved efficacy with potentially reduced off-target toxicity.

Substantial work remains before widespread clinical implementation of glycomic diagnostics and therapeutics. Analytical standardization, large prospective clinical validation studies, regulatory approval, cost reduction, and the development of accessible technologies are all necessary next steps. However, the scientific foundation is solid, the clinical need is urgent, and the potential for transforming women’s health outcomes is substantial. Investment in glycomic research, multidisciplinary collaboration, and translational research initiatives linking molecular discoveries to clinical applications will accelerate the integration of glycomics into precision medicine for gynecological diseases. As these advances progress over the next 2–3 years, gynecologic clinicians should anticipate that glycomic biomarkers will increasingly influence early detection algorithms, risk stratification, treatment selection, and treatment monitoring, fundamentally improving outcomes for women with gynecologic disorders globally.

## Figures and Tables

**Table 1:** Spectrum of Glycomic Dysregulation Across Gynecological Pathologies.

Disease State	Key Glycomic Alteration	Underlying Molecular Mechanism	Pathological Consequence
Gynecologic Malignancies (Ovarian, Cervical, Endometrial)	Increased N-glycan branching	Upregulation of GnT-V (MGAT5) creates structures that bind galectin-3.	Formation of surface lattices that sequester growth factor receptors, driving constitutive proliferative signaling.
	Hypersialylation	Excessive terminal sialic acid residues regulated by sialyltransferases (e.g., ST6GALNAC4).	Creates a “don’t eat me” glycan shield engaging Siglec receptors to suppress anti-tumor immunity.
	Truncated O-glycans	Epigenetic silencing of COSMC chaperone prevents O-glycan maturation, exposing Tn and sTn antigens.	Exposure of tumor-associated carbohydrate antigens (TACAs) facilitates immune evasion and metastasis.
Polycystic Ovary Syndrome (PCOS)	Altered Biosynthetic Precursors	Dysregulated glucose/lipid metabolism shifts nucleotide sugar substrate availability (UDP-GlcNAc, CMP-sialic acid).	Systematic reshaping of the serum glycome due to altered metabolic flux toward glycolytic intermediates.
	Inflammation-Associated Sialylation	Elevated cytokines (TNF-α, IL-6) upregulate sialyltransferases.	Increased sialylation of circulating proteins serving as potential biomarkers for disease severity.
Endometriosis	Dysregulated Estrogen Signaling	Reduced expression of ZMIZ1 (coregulator of ESR1) alters glycosyltransferase transcription.	Aberrant glycosylation of proteins critical for cell-cell adhesion and immune recognition in eutopic endometrium.
Infertility/Implantation Failure	Endometrial Glycosylation Defects	Altered glycosylation of adhesion molecules, cytokines, and follistatin.	Impaired embryo-endometrial interactions and uterine receptivity defects leading to implantation failure.

**Table 2:** Emerging Glycan-Targeting Therapeutic Strategies in Women’s Health.

Therapeutic Modality	Glycan/Molecule Target	Mechanism of Action	Clinical/Preclinical Insight
Antibody–Drug Conjugates (ADCs)	Sialyl-Tn (sTn)	Delivers cytotoxic payloads specifically to cells expressing tumor-restricted glycan epitopes.	sTn is abundant in gynecologic malignancies but essentially absent in healthy tissue, minimizing off-target toxicity; currently in clinical trials.
CAR-NK/CAR-T Cells	Tn-MUC1	Engineered immune cells recognize glycopeptide epitopes (incomplete O-glycosylation on mucins).	CAR-NK cells offer an “off-the-shelf” allogeneic option; demonstrates potential for durable anti-tumor response and immune memory.
Sugar-Stripping Enzymes	Terminal Sialic Acids	Sialidase fusion proteins enzymatically remove the sialic acid “shield” from tumor surfaces.	Unmasks hidden tumor antigens and prevents Siglec-mediated immune suppression; shows synergy with anti-PD-1 checkpoint inhibitors.
Glycosyltransferase Inhibitors	Sialyltransferases (e.g., ST6GALNAC4)	Direct small-molecule inhibition of enzymes driving hypersialylation.	Potential to reduce immune evasion driven by MYC pathways; requires high selectivity to avoid disrupting healthy tissue glycosylation.
Fucosyltransferase Inhibitors	Fucosyltransferases	Inhibition of enzymes responsible for adding fucose residues to glycans.	May reduce fucosylation-driven epithelial-to-mesenchymal transition (EMT) and metastatic dissemination in adenocarcinomas

## Data Availability

No new data were created or analyzed in this study. Data sharing is not applicable to this article.

## References

[1] Yang Q. (2025). Uncovering the Cellular and Molecular Landscape of Gynecological Disorders. Cells.

[2] Singh S., Pal N., Shubham S., Sarma D.K., Verma V., Marotta F., Kumar M. (2023). Polycystic Ovary Syndrome: Etiology, Current Management, and Future Therapeutics. J. Clin. Med..

[3] Parasar P., Ozcan P., Terry K.L. (2017). Endometriosis: Epidemiology, Diagnosis and Clinical Management. Curr. Obstet. Gynecol. Rep..

[4] Zhu B., Gu H., Mao Z., Beeraka N.M., Zhao X., Anand M.P., Zheng Y., Zhao R., Li S., Manogaran P. (2024). Global burden of gynaecological cancers in 2022 and projections to 2050. J. Glob. Health.

[5] Kim B., Park Y., Kim B., Ahn H.J., Lee K.A., Chung J.E., Han S.W. (2019). Diagnostic performance of CA 125, HE4, and risk of Ovarian Malignancy Algorithm for ovarian cancer. J. Clin. Lab. Anal..

[6] Rosendo-Chalma P., Díaz-Landy E.N., Antonio-Véjar V., Ortiz Tejedor J.G., Reytor-González C., Simancas-Racines D., Bigoni-Ordóñez G.D. (2025). Endometriosis: Challenges in Clinical Molecular Diagnostics and Treatment. Int. J. Mol. Sci..

[7] Staudacher E., Van Damme E.J.M., Smagghe G. (2022). Glycosylation-The Most Diverse Post-Translational Modification. Biomolecules.

[8] Kailemia M.J., Park D., Lebrilla C.B. (2017). Glycans and glycoproteins as specific biomarkers for cancer. Anal. Bioanal. Chem..

[9] Dos Reis J.S., Rodrigues da Costa Santos M.A., Mendonça D.P., Martins do Nascimento S.I., Barcelos P.M., Correia de Lima R.G., da Costa K.M., Freire-de-Lima C.G., Morrot A., Previato J.O. (2022). Glycobiology of Cancer: Sugar Drives the Show. Medicines.

[10] Shen H., Lee C.-Y., Chen C.-H. (2022). Protein Glycosylation as Biomarkers in Gynecologic Cancers. Diagnostics.

[11] Ruhaak L.R., Xu G., Li Q., Goonatilleke E., Lebrilla C.B. (2018). Mass Spectrometry Approaches to Glycomic and Glycoproteomic Analyses. Chem. Rev..

[12] Trbojević-Akmačić I., Lageveen-Kammeijer G.S.M., Heijs B., Petrović T., Deriš H., Wuhrer M., Lauc G. (2022). High-Throughput Glycomic Methods. Chem. Rev..

[13] Ohtsubo K., Marth J.D. (2006). Glycosylation in Cellular Mechanisms of Health and Disease. Cell.

[14] Breitling J., Aebi M. (2013). N-linked protein glycosylation in the endoplasmic reticulum. Cold Spring Harb. Perspect. Biol..

[15] Bieberich E. (2014). Synthesis, Processing, and Function of N-glycans in N-glycoproteins. Adv. Neurobiol..

[16] Hanisch F.-G. (2001). O-Glycosylation of the Mucin Type. Biol. Chem..

[17] Ju T., Wang Y., Aryal R.P., Lehoux S.D., Ding X., Kudelka M.R., Cutler C., Zeng J., Wang J., Sun X. (2013). Tn and sialyl-Tn antigens, aberrant O-glycomics as human disease markers. Proteom. Clin. Appl..

[18] Lagana A., Goetz J.G., Cheung P., Raz A., Dennis J.W., Nabi I.R. (2006). Galectin binding to Mgat5-modified N-glycans regulates fibronectin matrix remodeling in tumor cells. Mol. Cell. Biol..

[19] Chen Q.-W., Zhang Y., Bao P. (2024). Sialidase-Chimeric Bioengineered Bacteria for Tumor-Sialoglycan-Triggered Solid Tumor Therapy. Nano Lett..

[20] Radhakrishnan P., Dabelsteen S., Madsen F.B., Francavilla C., Kopp K.L., Steentoft C., Vakhrushev S.Y., Olsen J.V., Hansen L., Bennett E.P. (2014). Immature truncated O-glycophenotype of cancer directly induces oncogenic features. Proc. Natl. Acad. Sci. USA.

[21] Dong J., Rees D.A. (2023). Polycystic ovary syndrome: Pathophysiology and therapeutic opportunities. BMJ Med..

[22] Holman M., Li S.J., Ahern M.M., Ruhaak L.R., Karakas S., Krishnan S. (2025). The Plasma Glycome of Women with PCOS is Different from Healthy Controls. medRxiv.

[23] Yan H., Wang L., Zhang G., Li N., Zhao Y., Liu J., Jiang M., Du X., Zeng Q., Xiong D. (2024). Oxidative stress and energy metabolism abnormalities in polycystic ovary syndrome: From mechanisms to therapeutic strategies. Reprod. Biol. Endocrinol..

[24] Zhu R., Yu X., Li Y. (2025). Identification and validation of biomarkers associated with glycolysis in polycystic ovarian syndrome. Sci. Rep..

[25] Czubak P., Herda K., Niewiadomska I., Putowski L., Łańcut M., Masłyk M. (2025). Understanding Endometriosis: A Broad Review of Its Causes, Management, and Impact. Int. J. Mol. Sci..

[26] Rahman M.S., So K.A., Jeong J.W. (2025). ZMIZ1 and estrogen receptor α form an essential partnership in endometrial biology. J. Clin. Investig..

[27] Zhang L., Feng Y., Zhang Y., Sun X., Ma Q., Ma F. (2024). The Sweet Relationship between the Endometrium and Protein Glycosylation. Biomolecules.

[28] Anastasiu C.V., Moga M.A., Elena Neculau A., Bălan A., Scârneciu I., Dragomir R.M., Dull A.-M., Chicea L.-M. (2020). Biomarkers for the Noninvasive Diagnosis of Endometriosis: State of the Art and Future Perspectives. Int. J. Mol. Sci..

[29] Peng W., Zhao J., Dong X., Banazadeh A., Huang Y., Hussien A., Mechref Y. (2018). Clinical application of quantitative glycomics. Expert Rev. Proteom..

[30] Wanyama F.M., Blanchard V. (2021). Glycomic-Based Biomarkers for Ovarian Cancer: Advances and Challenges. Diagnostics.

[31] Saad A.A. (2022). Targeting cancer-associated glycans as a therapeutic strategy in leukemia. All Life.

[32] Gao Z., Xu M., Yue S., Shan H., Xia J., Jiang J., Yang S. (2022). Abnormal sialylation and fucosylation of saliva glycoproteins: Characteristics of lung cancer-specific biomarkers. Curr. Res. Pharmacol. Drug Discov..

[33] Leiserowitz G.S., Lebrilla C., Miyamoto S., An H.J., Duong H., Kirmiz C., Li B., Liu H., Lam K.S. (2008). Glycomics analysis of serum: A potential new biomarker for ovarian cancer?. Int. J. Gynecol. Cancer Off. J. Int. Gynecol. Cancer Soc..

[34] Martins Á.M., Ramos C.C. (2021). Glycosylation of Cancer Extracellular Vesicles: Capture Strategies, Functional Roles and Potential Clinical Applications. Cells.

[35] Escrevente C., Grammel N., Kandzia S., Zeiser J., Tranfield E.M., Conradt H.S., Costa J. (2013). Sialoglycoproteins and N-Glycans from Secreted Exosomes of Ovarian Carcinoma Cells. PLoS ONE.

[36] Jamali Z., Razipour M., Zargar M., Ghasemnejad-Berenji H., Akrami S.M. (2025). Ovarian cancer extracellular vesicle biomarkers. Clin. Chim. Acta Int. J. Clin. Chem..

[37] Zhang Y., Qin Q. (2025). Prospects and challenges of deep learning in gynecologic malignancies. Front. Oncol..

[38] Li M., Xie S., Lu C., Zhu L., Zhu L. (2021). Application of Data Science in Circulating Tumor DNA Detection: A Promising Avenue Towards Liquid Biopsy. Front. Oncol..

[39] Englisz A., Smycz-Kubańska M., Królewska-Daszczyńska P., Błaut M., Duszyc A., Mielczarek-Palacz A. (2025). The Application of Circulating Tumour DNA (ctDNA) in the Diagnosis, Prognosis, and Treatment Monitoring of Gynaecological and Breast Cancers (Review). Diagnostics.

[40] Sun S., Liu Y., Li L., Xiong L., Jiao M., Yang J., Li X., Liu W. (2024). Unveiling the shared genetic architecture between testosterone and polycystic ovary syndrome. Sci. Rep..

[41] Venturella R., Lico D., Sarica A., Falbo M.P., Gulletta E., Morelli M., Zupi E., Cevenini G., Cannataro M., Zullo F. (2015). OvAge: A new methodology to quantify ovarian reserve combining clinical, biochemical and 3D-ultrasonographic parameters. J. Ovarian Res..

[42] Wu C., Sun Y., Yang D., Peng H. (2025). Advances in endometrial receptivity and embryo implantation by multi-omics techniques. Anim. Zoonoses.

[43] Guzeloglu-Kayisli O., Kayisli U.A., Taylor H.S. (2009). The role of growth factors and cytokines during implantation: Endocrine and paracrine interactions. Semin. Reprod. Med..

[44] Sun X., Feng Y., Ma Q., Wang Y., Ma F. (2023). Protein Glycosylation, Bridging Maternal-Fetal Crosstalk during Embryo Implantation. Biol. Reprod..

[45] Fullerton P.T., Monsivais D., Kommagani R., Matzuk M.M. (2017). Follistatin is critical for mouse uterine receptivity and decidualization. Proc. Natl. Acad. Sci. USA.

[46] Melchiorre C., Chhuon C., Jung V., Lipecka J., Di Rella F., Conforti A., Amoresano A., Carpentieri A., Guerrera I.C. (2021). Identification and Relative Quantification of hFSH Glycoforms in Women’s Sera via MS-PRM-Based Approach. Pharmaceutics.

[47] Gui N., Cheewakriangkrai C., Chaiyawat P., Udomruk S. (2025). Unlocking the Potential of Liquid Biopsy: A Paradigm Shift in Endometrial Cancer Care. Diagnostics.

[48] Peng W., Gutierrez Reyes C.D., Gautam S., Yu A., Cho B.G., Goli M., Donohoo K., Mondello S., Kobeissy F., Mechref Y. (2023). MS-based glycomics and glycoproteomics methods enabling isomeric characterization. Mass Spectrom. Rev..

[49] Powers T.W., Jones E.E., Betesh L.R., Romano P.R., Gao P., Copland J.A., Mehta A.S., Drake R.R. (2013). Matrix assisted laser desorption ionization imaging mass spectrometry workflow for spatial profiling analysis of N-linked glycan expression in tissues. Anal. Chem..

[50] Xu W., Lu J., Zhang H., Ye D. (2025). Decoding the tumor microenvironment: Insights into immunotherapy and beyond. J. Natl. Cancer Cent..

[51] Abu Bakar N., Hamzan N.I. (2025). Advancement in Clinical Glycomics and Glycoproteomics for Congenital Disorders of Glycosylation: Progress and Challenges Ahead. Biomedicines.

[52] Vesper H.W., Myers G.L., Miller W.G. (2016). Current practices and challenges in the standardization and harmonization of clinical laboratory tests. Am. J. Clin. Nutr..

[53] Patabandige M.W., Pfeifer L.D., Nguyen H.T., Desaire H. (2022). Quantitative clinical glycomics strategies: A guide for selecting the best analysis approach. Mass Spectrom. Rev..

[54] Ampofo I., Takyi E., Kusi B., Nsobeah L., Foriwaa M., Adjei P., Ampofo I., Ampofo B. (2025). Machine Learning Algorithms for Diseases Prediction: A Systematic Review. Intelligent Sustainable Systems.

[55] Zhu X., Zhao Y., Zan C., Ma H., Liu J. (2025). Recent advances in applications of artificial intelligence-assisted Raman spectroscopy in diagnosis of cancers. Front. Mol. Biosci..

[56] Abbas Q., Jeong W., Lee S.W. (2025). Explainable AI in Clinical Decision Support Systems: A Meta-Analysis of Methods, Applications, and Usability Challenges. Healthcare.

[57] Adler-Milstein J., Aggarwal N., Ahmed M., Castner J., Evans B.J., Gonzalez A.A., James C.A., Lin S., Mandl K.D., Matheny M.E. (2022). Meeting the Moment: Addressing Barriers and Facilitating Clinical Adoption of Artificial Intelligence in Medical Diagnosis. NAM Perspect..

[58] Liu G., Yang S., Li J., Zheng Z., Zhang C., He Y., Wang Y., Kang W., Ye X. (2025). Biomarker-based and interpretable machine learning framework for predicting pathological stage in gastric cancer: A retrospective analysis. Digit. Health.

[59] Zheng M., Hu Y., Gou R., Wang J., Nie X., Li X., Liu Q., Liu J., Lin B. (2019). Integrated multi-omics analysis of genomics, epigenomics, and transcriptomics in ovarian carcinoma. Aging.

[60] Boyarskikh U.A., Gulyaeva L.F., Avdalyan A.M., Kechin A.A., Khrapov E.A., Lazareva D.G., Kushlinskii N.E., Melkonyan A., Arakelyan A., Filipenko M.L. (2020). Spectrum of TP53 Mutations in BRCA1/2 Associated High-Grade Serous Ovarian Cancer. Front. Oncol..

[61] Navarro Quiroz E., Chavez-Estrada V., Macias-Ochoa K., Ayala-Navarro M.F., Flores-Aguilar A.S., Morales-Navarrete F., de la Cruz Lopez F., Gomez Escorcia L., Musso C.G., Aroca Martinez G. (2019). Epigenetic Mechanisms and Posttranslational Modifications in Systemic Lupus Erythematosus. Int. J. Mol. Sci..

[62] Vaghani B. (2020). Integrating multi-omics data for early cancer detection: A machine learning framework for risk stratification. Int. J. Med. Health Res..

[63] Thurin M. (2021). Tumor-Associated Glycans as Targets for Immunotherapy: The Wistar Institute Experience/Legacy. Monoclon. Antibodies Immunodiagn. Immunother..

[64] Prendergast J.M., Galvao da Silva A.P., Eavarone D.A., Ghaderi D., Zhang M., Brady D., Wicks J., DeSander J., Behrens J., Rueda B.R. (2017). Novel anti-Sialyl-Tn monoclonal antibodies and antibody-drug conjugates demonstrate tumor specificity and anti-tumor activity. mAbs.

[65] Jiang Y., Xu Y., He J., Sui L., Li T., Xia N., Yao Q. (2025). Uncovering potential targets for antibody-drug conjugates in the treatment of gynecologic malignancies. Front. Pharmacol..

[66] Rafei H., Daher M., Rezvani K. (2021). Chimeric antigen receptor (CAR) natural killer (NK)-cell therapy: Leveraging the power of innate immunity. Br. J. Haematol..

[67] Posey AD J.r., Schwab R.D., Boesteanu A.C., Steentoft C., Mandel U., Engels B., Stone J.D., Madsen T.D., Schreiber K., Haines K.M. (2016). Engineered CAR T Cells Targeting the Cancer-Associated Tn-Glycoform of the Membrane Mucin MUC1 Control Adenocarcinoma. Immunity.

[68] Peng L., Sferruzza G., Yang L., Zhou L., Chen S. (2024). CAR-T and CAR-NK as cellular cancer immunotherapy for solid tumors. Cell. Mol. Immunol..

[69] Ghasempour S., Freeman S. (2021). The glycocalyx and immune evasion in cancer. FEBS J..

[70] Garabedian B.M., Bashian E.E., Wang X., Thompson A.J., Paulson J.C. (2025). Targeting Sialidase to PD1 Enhances T cell Function and Tumor Control. ACS Cent. Sci..

[71] Berckmans Y., Ceusters J., Vankerckhoven A., Wouters R., Riva M., Coosemans A. (2023). Preclinical studies performed in appropriate models could help identify optimal timing of combined chemotherapy and immunotherapy. Front. Immunol..

[72] Dalziel M., Crispin M., Scanlan C.N., Zitzmann N., Dwek R.A. (2014). Emerging Principles for the Therapeutic Exploitation of Glycosylation. Science.

[73] Dobie C., Skropeta D. (2021). Insights into the role of sialylation in cancer progression and metastasis. Br. J. Cancer.

[74] Ghirardello M., Yruela I., Merino P., Sackstein R., Sanz-Martínez I., Hurtado-Guerrero R. (2025). Structure, function, and implications of fucosyltransferases in health and disease. Nat. Commun..

[75] Edwards E., Livanos M., Krueger A., Dell A., Haslam S.M., Mark Smales C., Bracewell D.G. (2022). Strategies to control therapeutic antibody glycosylation during bioprocessing: Synthesis and separation. Biotechnol. Bioeng..

[76] Man D., Jiang Y., Zhang D., Wu J., Ding B., Liu H., Xu G., Lu J., Ru J., Tong R. (2023). ST6GALNAC4 promotes hepatocellular carcinogenesis by inducing abnormal glycosylation. J. Transl. Med..

[77] Wang Y., Lei K., Zhao L., Zhang Y. (2024). Clinical glycoproteomics: Methods and diseases. MedComm.

[78] Ruhaak L.R., Miyamoto S., Lebrilla C.B. (2013). Developments in the identification of glycan biomarkers for the detection of cancer. Mol. Cell. Proteom. MCP.

[79] Pothuri B., Thaker P., Moore A., Espinosa R., Medina K., Collyar D., Lutz K., Munteanu M.C., Slomovitz B. (2025). Improving diverse patient enrollment in clinical trials, focusing on Hispanic and Asian populations: Recommendations from an interdisciplinary expert panel. Int. J. Gynecol. Cancer Off. J. Int. Gynecol. Cancer Soc..

[80] Ki M.-R., Kim D.H., Abdelhamid M.A.A., Pack S.P. (2025). Cancer and Aging Biomarkers: Classification, Early Detection Technologies and Emerging Research Trends. Biosensors.

[81] Budelier M.M., Hubbard J.A. (2023). The regulatory landscape of laboratory developed tests: Past, present, and a perspective on the future. J. Mass Spectrom. Adv. Clin. Lab.

[82] Post M.A., Lefeber D.J. (2019). Clinical glycomics in the diagnostic laboratory. Ann. Transl. Med..

[83] Furukawa J., Fujitani N., Shinohara Y. (2013). Recent advances in cellular glycomic analyses. Biomolecules.

[84] Bhaiyya M., Panigrahi D., Rewatkar P., Haick H. (2024). Role of Machine Learning Assisted Biosensors in Point-of-Care-Testing For Clinical Decisions. ACS Sens..

